# Enterovirus 71 Binding to PSGL-1 on Leukocytes: VP1-145 Acts as a Molecular Switch to Control Receptor Interaction

**DOI:** 10.1371/journal.ppat.1003511

**Published:** 2013-07-25

**Authors:** Yorihiro Nishimura, Hyunwook Lee, Susan Hafenstein, Chikako Kataoka, Takaji Wakita, Jeffrey M. Bergelson, Hiroyuki Shimizu

**Affiliations:** 1 Department of Virology II, National Institute of Infectious Diseases, Musashimurayama-shi, Tokyo, Japan; 2 Division of Infectious Diseases, The Children's Hospital of Philadelphia, Philadelphia, Pennsylvania, United States of America; 3 Department of Microbiology and Immunology, Penn State University College of Medicine, Hershey, Pennsylvania, United States of America; 4 Department of Pediatrics, University of Pennsylvania Perelman School of Medicine, Philadelphia, Pennsylvania, United States of America; University of Pittsburgh, United States of America

## Abstract

Some strains of enterovirus 71 (EV71), but not others, infect leukocytes by binding to a specific receptor molecule: the P-selectin glycoprotein ligand-1 (PSGL-1). We find that a single amino acid residue within the capsid protein VP1 determines whether EV71 binds to PSGL-1. Examination of capsid sequences of representative EV71 strains revealed that the PSGL-1-binding viruses had either a G or a Q at residue 145 within the capsid protein VP1 (VP1-145G or Q), whereas PSGL-1-nonbinding viruses had VP1-145E. Using site-directed mutagenesis we found that PSGL-1-binding strains lost their capacity to bind when VP1-145G/Q was replaced by E; conversely, nonbinding strains gained the capacity to bind PSGL-1 when VP1-145E was replaced with either G or Q. Viruses with G/Q at VP1-145 productively infected a leukocyte cell line, Jurkat T-cells, whereas viruses with E at this position did not. We previously reported that EV71 binds to the N-terminal region of PSGL-1, and that binding depends on sulfated tyrosine residues within this region. We speculated that binding depends on interaction between negatively charged sulfate groups and positively charged basic residues in the virus capsid. VP1-145 on the virus surface is in close proximity to conserved lysine residues at VP1-242 and VP1-244. Comparison of recently published crystal structures of EV71 isolates with either Q or E at VP1-145 revealed that VP1-145 controls the orientation of the lysine side-chain of VP1-244: with VP1-145Q the lysine side chain faces outward, but with VP1-145E, the lysine side chain is turned toward the virus surface. Mutation of VP1-244 abolished virus binding to PSGL-1, and mutation of VP1-242 greatly reduced binding. We propose that conserved lysine residues on the virus surface are responsible for interaction with sulfated tyrosine residues at the PSGL-1 N-terminus, and that VP1-145 acts as a switch, controlling PSGL-1 binding by modulating the exposure of VP1-244K.

## Author Summary

Enterovirus 71 (EV71) commonly causes mild febrile illness in children (hand, foot, and mouth disease), but some patients suffer severe neurologic disease and death. Recent outbreaks in the Asia-Pacific region have caused thousands of deaths, making EV71 a major public health concern. Some EV71 strains bind to P-selectin glycoprotein ligand-1 (PSGL-1) and infect immune cells, but others do not. We previously found that EV71 binds the PSGL-1 N-terminus, and that binding depends on tyrosine sulfation of the N-terminus, but the viral factors that control interaction with PSGL-1 have not been identified. In our present work we present evidence that a single amino acid, residue 145 of the viral capsid protein (VP1-145), determines whether a virus binds or does not bind PSGL-1, and that it functions by influencing the orientation of a nearby lysine residue (VP1-244) on the virus surface. We propose that VP1-145 controls virus tropism by changing the accessibility of the positively-charged lysine side chain of VP1-244 to the negatively charged, sulfated N-terminus of PSGL-1. Our results shed new light on virus-receptor interaction, and EV71 tropism for PSGL-1-expressing leukocytes.

## Introduction

Enterovirus 71 (EV71) is a small, non-enveloped positive-stranded RNA virus that belongs to the human enterovirus species A of the genus *Enterovirus* in the family *Picornaviridae* [1]. The viral RNA genome is enclosed in a capsid composed of four structural proteins, VP1, VP2, VP3, and VP4 [2], [3], [4]. EV71 is a major causative agent of hand, foot, and mouth disease (reviewed in [5], [6], [7], [8]), a febrile illness that commonly affects young children. Although hand, foot, and mouth disease is usually mild and self-limited, EV71 infection may also cause severe diseases including poliomyelitis-like paralysis, brainstem encephalitis, and fatal cardiorespiratory failure. Recent EV71 outbreaks in the Asia-Pacific region have involved millions of children, and have caused thousands of deaths [9], [10].

Recently, several cell-surface molecules have been identified to be involved in EV71 infection [11], [12], [13], [14], [15], [16]. EV71 isolates use at least two transmembrane proteins as receptors (reviewed in [17], [18]). Scavenger receptor class B, member 2 (SCARB2), originally identified as an EV71 receptor on rhabdomyosarcoma (RD) cells [14], is expressed on a broad variety of cell types. In contrast, P-selectin glycoprotein ligand-1 (PSGL-1), which we first identified as an EV71 receptor on Jurkat T cells [12], is primarily expressed on leukocytes, where it mediates interaction with selectins and thus serves an important function in inflammatory processes [19], [20]. Whereas SCARB2 serves as a receptor for all EV71 strains tested, as well as for several other viruses (coxsackieviruses A7, A14, and A16) that are not associated with severe disease [21], PSGL-1 interacts with a distinct subset of EV71 strains. According to their PSGL-1-binding capacity, EV71 strains can be classified into two distinct phenotypes— irrespective of their genogroups— which we designate as PSGL-1-binding (PB) and PSGL-1-nonbinding (non-PB) strains [12]. PB viruses bind to PSGL-1 to replicate in Jurkat cells, and their replication in Jurkat cells is blocked by an anti-PSGL-1 monoclonal antibody (mAb). Non-PB viruses either fail to replicate in Jurkat cells or replicate in a PSGL-1-independent manner. In RD cells, which express SCARB2, both PB and non-PB isolates replicate independently of PSGL-1.

We previously showed that the N-terminal region of human PSGL-1 (amino acids 42–61) is directly responsible for PSGL-1 binding to EV71 [12]. This N-terminal region is also critical for PSGL-1 binding to P-, E- and L-selectins. Post-translational modifications of the N-terminal region— including tyrosine sulfation and *O*-glycosylation— are important for recognition by selectins [22], [23], [24], [25]. We have demonstrated that sulfation of three tyrosines (Y46, Y48, and Y51) is essential for binding of EV71 to PSGL-1 [26]. We therefore suspected that these negatively charged residues might promote virus binding by interacting with positively charged residues on the virus surface.

We have now used a combination of mutational and structural analysis to clarify the molecular basis for EV71 interaction with PSGL-1. We find that a single amino acid residue, VP1-145, regulates binding to PSGL-1 by changing the orientation of a critical lysine residue on the virus surface.

## Results

### Comparison of PB and non-PB isolates reveals just two consistent amino acid differences within the capsid region

To identify potential genetic determinants of PSGL-1 binding we compared the capsid sequences of EV71 strains (listed in Table 1) that we had previously characterized as PB or non-PB [12], [27]. EV71 isolates are classified into three genogroups (A, B, and C) according to their VP1 nucleotide sequences [28], and genogroups B and C are each further divided into five subgenogroups [29]. We began our analysis by comparing the capsid amino acid sequences of two viruses in subgenogroup C1, EV71-KED005 (PB) and -02363 (non-PB). There were only four differences (VP3-55, VP1-98, VP1-145, and VP1-262) within the 862 amino acid capsid region (Table 1), as we previously reported [12]. The amino acid at VP1-145 of EV71-KED005 could not be determined because there was a mixture of sequences at this position [27].

**Table 1:** Representation among EV71 isolates of the four amino acids found to differ between KED005 and 02363.

Strain (Subgenogroup)	Accession No.	PB phenotype1)	VP3-55	VP1-98	VP1-145	VP1-262
C7/Osaka (B3) [54]	AB550336	PB	V	E	G	I
SK-EV006 (B4) [54]	AB550334	PB	V	E	G	I
1095 (C2) [56], [57]	AB550332	PB	V	E	G	I
75-Yamagata (C4) [55]	AB550338	PB	V	E	Q	I
KED005 (C1) [54]	AB550340	PB	V	E	n.d.2)	V
02363 (C1) [57]	AB747375	Non-PB	I	K	E	I
BrCr (A) [53]	AB777928	Non-PB	V	K	E	I
Nagoya (B1) [58]	AB747373	Non-PB	V	E	E	I

1) PB: PSGL-1-binding, Non-PB: PSGL-1-nonbinding [12].

2) Not determined as there was a mixture of sequences at this position.

We then examined the sequences of all eight strains, noting the amino acids at VP3-55, VP1-98, VP1-145, and VP1-262 (Table 1). We found a strong relationship between the PSGL-1 binding phenotype and the specific amino acids at VP1-98 and VP1-145: the five PB strains showed a combination of VP1-98E and VP1-145G (which we designated EG), or VP1-98E and VP1-145Q (EQ), whereas non-PB strains showed VP1-98K and VP1-145E (KE), or VP1-98E and VP1-145E (EE). The amino acids at VP3-55 and VP1-262 did not correlate with the PSGL-1 binding phenotypes. Thus we focused on VP1-98 and VP1-145 as possible determinants of PSGL-1 binding.

### EV71 strains can be classified into four major groups according to the amino acids at VP1-98 and VP1-145

The eight virus isolates we examined above showed four combinations of amino acids at VP1-98 and VP1-145: EG, EQ, EE and KE. To determine how frequently these combinations are found in other isolates, we examined EV71 nucleotide sequences available in GenBank database [as of January 2011, 1702 sequences included codons for both VP1-98 and VP1-145 (Table S1)]. Interestingly, we found one of the same four combinations of amino acids in virtually all the available sequences (Table 2). The EE was seen in 71% of isolates, and the other combinations were each seen in approximately 9%. We focused on these four amino acid combinations for further investigation, as they accounted for 98.8% of the total strains.

**Table 2:** Amino acid combinations at VP1-98 and VP1-145 in 1702 EV71 sequences found in GenBank.

	VP1-145						
VP1-98	G	Q	E	A	R	K	n.d1)
E	156 (9.2)	155 (9.1)	1209 (71.0)	5 (0.3)	2 (0.1)	5 (0.3)	2 (0.1)
K	0	0	161 (9.5)	0	1 (0.1)	0	0
Q	0	0	2 (0.1)	0	0	0	0
N	0	0	1 (0.1)	0	0	0	0
G	0	0	1 (0.1)	0	0	0	0
n.d.*	0	0	2 (0.1)	0	0	0	0

The percentage in 1702 sequences is shown in parentheses.

1) Not determined as there was a mixture of sequences at this position.

### Production of cDNA-derived EV71

To determine the amino acids that account for the PSGL-1 binding phenotype, we generated infectious cDNA clones with amino acid mutations at VP1-98 and/or VP1-145. We cloned full-length genomic cDNA of five strains (C7/Osaka, Nagoya, 1095, 02363, and 75-Yamagata) into pBR322Y plasmids. The cDNA clones of C7/Osaka, 1095, 02363, and 75-Yamagata were corrected to match the consensus sequences that had been determined by direct sequencing of reverse transcription polymerase chain reaction (RT-PCR) products. The cDNA clone of Nagoya (GenBank Accession No. AB747373) was not corrected, and differed from the full-length consensus nucleotide sequence (GenBank Accession No. AB747373) at eight positions (Y930C, Y933C, Y1662C, S2005G, S2416C, Y3547T, W3602A, and Y4038T).

**Figure 1:**
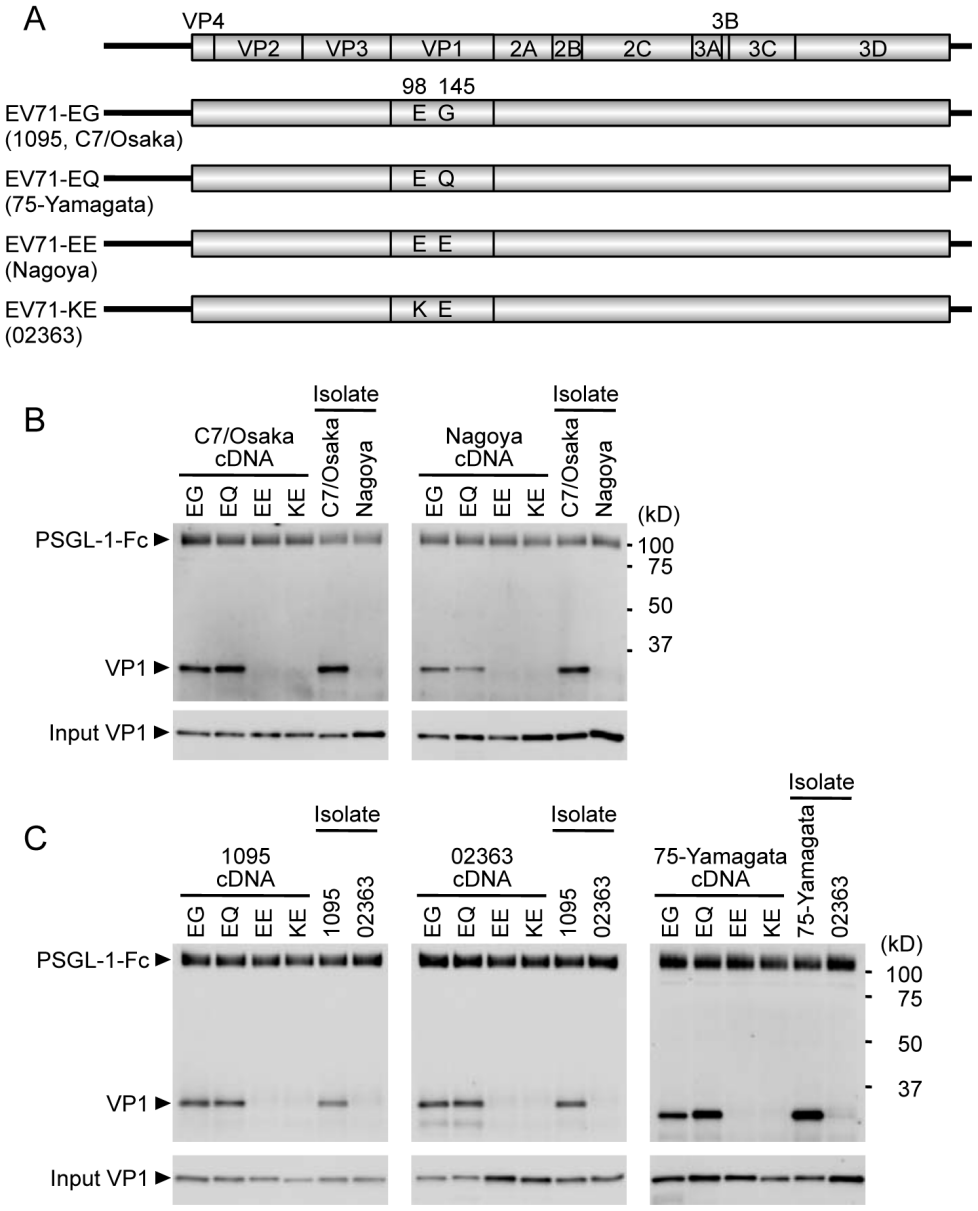
Co-precipitation analysis of EV71 mutants with soluble PSGL-1-Fc. (A) Scheme of EV71 constructs with amino acid substitutions at VP1-98 and VP1-145. The original strains, which have the indicated amino acids at VP1-98 and VP1-145, are indicated in parentheses. (B, C) The co-precipitation of EV71 and PSGL-1-Fc was detected by western blotting using anti-VP1 mAb and an anti-Fc antibody. EV71-PB (C7-Osaka, 1095, 75-Yamagata) and EV71-non-PB (Nagoya and 02363) isolates (not derived from cDNA) were used as positive and negative controls for binding to PSGL-1-Fc, respectively. The data are representative of three independent experiments. (B) EV71 strains of genogroup B. (C) EV71 strains of genogroup C.

Into each of the cloned viral genomes we introduced mutations to produce each of the four major combinations of residues at VP1-98 and VP1-145 (residues at these positions in the original isolates are shown in Figure 1A). Viral RNAs were transcribed *in vitro* using T7 RNA polymerase, and transfected into RD cells to produce viruses, which were collected at 24 h post-transfection and amplified once in fresh RD cells. The capsid-encoding regions of each of the amplified virus mutants were determined by direct sequencing of RT-PCR products. The sequences of 18 of the 20 amplified viruses (apart from codons for VP1-98 and 145) were identical to those of the cDNA clones. Amplified Nagoya-EG virus had a single nucleotide substitution, C3152A, which resulted in an amino acid change at VP1-T237N; this mutation is unlikely to affect virus interaction with PSGL-1, as VP1-237 is located in the βH barrel, not in the most exposed loops [2], [3], [4]. Amplified Yamagata-EE had a silent nucleotide substitution, C3054T, which would not affect virus interaction with PSGL-1.

### VP1-145 controls virus binding to PSGL-1

We examined the direct biochemical interaction between EV71 mutants and PSGL-1 by co-precipitating viruses with a soluble form of recombinant PSGL-1 fused to the Fc region of human IgG_1_ (PSGL-1-Fc), as described previously [12]. EV71 that co-precipitated with PSGL-1-Fc was detected by western blotting with an anti-VP1 mAb.

We first tested mutants derived from genogroup B strains (Figure 1B), to determine whether mutation at VP1-98 or VP1-145 abolished PSGL-1 binding by the PB strain (C7/Osaka), or conferred PSGL-1 binding capacity on the non-PB strain (Nagoya). We used the original isolates of C7/Osaka and Nagoya (not produced from cDNA clones) as positive and negative controls, respectively. In either the C7/Osaka or the Nagoya background, viruses with EG or EQ, but not EE or KE, immunoprecipitated with PSGL-1-Fc. Thus, the presence of G or Q at VP1-145, but not E, was associated with binding to PSGL-1 in these two genogroup B strains; the presence of K or E at VP1-98 did not influence binding. Similar results were obtained with the genogroup C strains 1095 (PB) and 02363 (non-PB) and with 75-Yamagata, a PB strain which normally has a Q at VP1-145 (Figure 1C): in each case, viruses with G or Q— but not those with E— at VP1-145 bound PSGL-1, irrespective of the residue at VP1-98. We also substituted VP1-145 with several amino acids found less commonly in GenBank (Table 2). EV71-1095 with VP1-145A bound to PSGL-1 as had been previously observed for another viral strain [30]. VP1-145R similarly bound to PSGL-1; however, virus with K or D at this position showed little or no binding to PSGL-1 (Figure S1). In the presence of VP1-145G or Q, viruses with either VP1-98E or VP1-98K bound to PSGL-1-Fc (KG and KQ in Figure S1). Taken together, these results suggested that the identity of VP1-145 regulates the capacity of EV71 to bind PSGL-1.

### VP1-145 controls PSGL-1-dependent replication of EV71 in Jurkat cells

**Figure 2:**
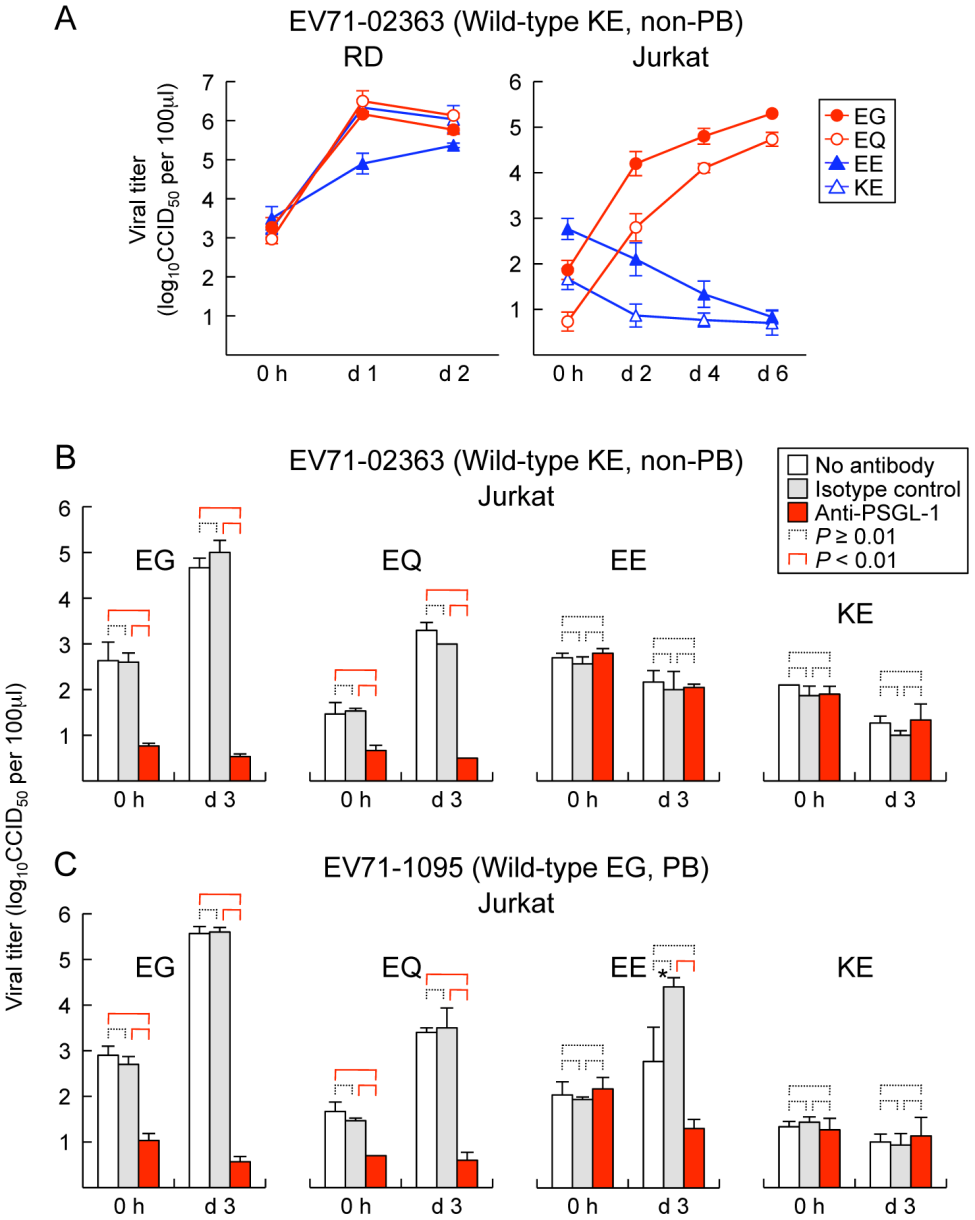
Replication of EV71 mutants in RD and Jurkat cells. (A) RD (left) or Jurkat (right) cells were infected with EV71-02363 mutants at 1 CCID_50_ per cell for 1 h, then washed, cultured, and harvested at the indicated days (d) post-infection. (B, C) EV71 replication in Jurkat cells incubated with anti-PSGL-1 mAb and isotype control. Cells were pretreated with antibodies for 1 h and then infected with 1 CCID_50_ per cell for 1 h, washed, cultured, and harvested at 3 days (d) post-infection. Viral titers are expressed as the mean, and error bars indicate s. d. for triplicate samples. (B) EV71-02363. (C) EV71-1095. Although arise in viral titer was observed for EV71-1095-EE at day 3 (asterisk), the recovered viruses were VP1-145E to G revertants.

We previously found that PB viruses productively infected Jurkat T lymphocytes, and that their replication was inhibited by anti-PSGL-1 mAb [12]. To determine whether mutations at VP1-145 influenced virus tropism for Jurkat cells, we examined replication in Jurkat cells of the wild-type and mutant derivatives of EV71-02363 (non-PB) and EV71-1095 (PB). Although all of the cDNA-derived 02363 mutants replicated well in RD cells (Figure 2A, left), no replication was seen with either of the non-PB mutants, either EV71-02363-EE and EV71-02363-KE in Jurkat cells (Figure 2A, right). In contrast, EV71-02363-EG and EV71-02363-EQ replicated well in Jurkat cells, and their replication was inhibited by anti-PSGL-1 mAb (Figure 2B). Thus, a non-PB isolate of EV71, which did not replicate in Jurkat cells, gained the capacity to replicate in a PSGL-1-dependent manner when VP1-145E was replaced by either G or Q. Conversely, when mutants of 1095 (EG, a PB strain) were tested, viruses with G or Q at VP1-145 replicated to high titer, but the virus with E at this position did not (Figure 2C); although some apparent replication was noted for the 1095-EE mutant, replication was inhibited by anti-PSGL-1 mAb, and sequencing of the recovered virus revealed a reversion of E to G at VP1-145 (not shown), suggesting that replication in Jurkat cells selected for a minor population of PB virus. Taken together with results shown in Figure 1, the data shown in Figure 2 indicate that the presence of a G or Q —rather than an E— at VP1-145 determines both virus binding to PSGL-1 and PSGL-1-dependent virus tropism for Jurkat cells.

We previously reported that EV71-1095 replicates to a limited degree in U937 monocytes as well as in Jurkat cells [12]. To test the role of VP1-145 in this interaction, we exposed U937 cells to PB (EG and EQ) and non-PB (EE and KE) derivatives of EV71-1095 and measured virus titers over 4 days. The PB, but not the PB derivatives showed a small but significant increase in titer (data not shown), confirming that VP145G/Q is a determinant of productive infection in this cell line. In contrast, PB derivatives of another virus isolate, EV71-02363, did not appear to replicate in U937 cells. Thus, viral factors other than the capacity to bind PSGL-1 are also likely to be important for productive infection in some leukocytes.

### VP1-145 is in close proximity to a lysine residue at VP1-244 on the virus surface

**Figure 3:**
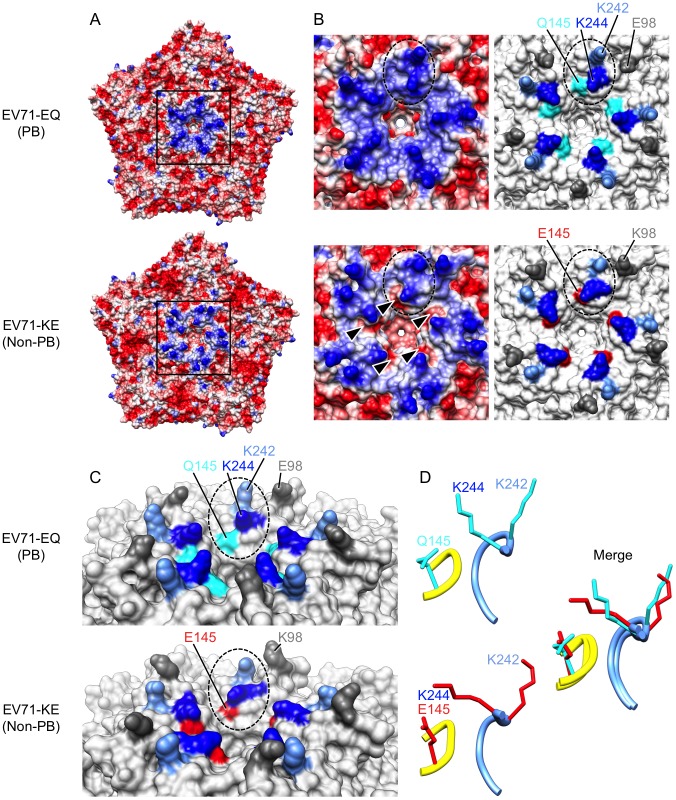
Comparison of EV71-PB and EV71-non-PB structures. Molecular surfaces of EV71-EQ (4AED; presumably PB, top of each figure section) and EV71-KE (3VBS; presumably non-PB, bottom of each figure section). (A) Electrostatic surface coloring of five icosahedral asymmetric units using UCSF Chimera software with ‘coulombic surface coloring’ function. Negatively charged surfaces are shown in red, whereas positively charged areas are shown in blue. Black-boxed areas are enlarged and shown in (B). (B) Molecular surfaces around the five-fold axis of symmetry. Dotted ovals indicate a set of VP1-145, VP1-242, and VP1-244. Left panels show electrostatic surface coloring. Arrowheads in EV71-KE show negatively charged patches around VP1-145E. Right panels show the positions of specific residues (dim gray, VP1-98; cyan, VP1-145Q; red, VP1-145E; cornflower blue, VP1-242K; blue, VP1-244K). (C) Oblique views of the five-fold axis. Dotted ovals indicate a set of VP1-145, VP1-242, and VP1-244. Specific residues are colored as in (B). (D) Loop structures of partial VP1-DE (yellow, VP1-142 to -145) and partial VP1-HI loops (cornflower blue, VP1-238 to -244) depicted in ribbon form. Side chains for the residues VP1-145, VP1-242, and VP1-244 are displayed as sticks (cyan, EV71-EQ; red, EV71-KE).

**Figure 4:**
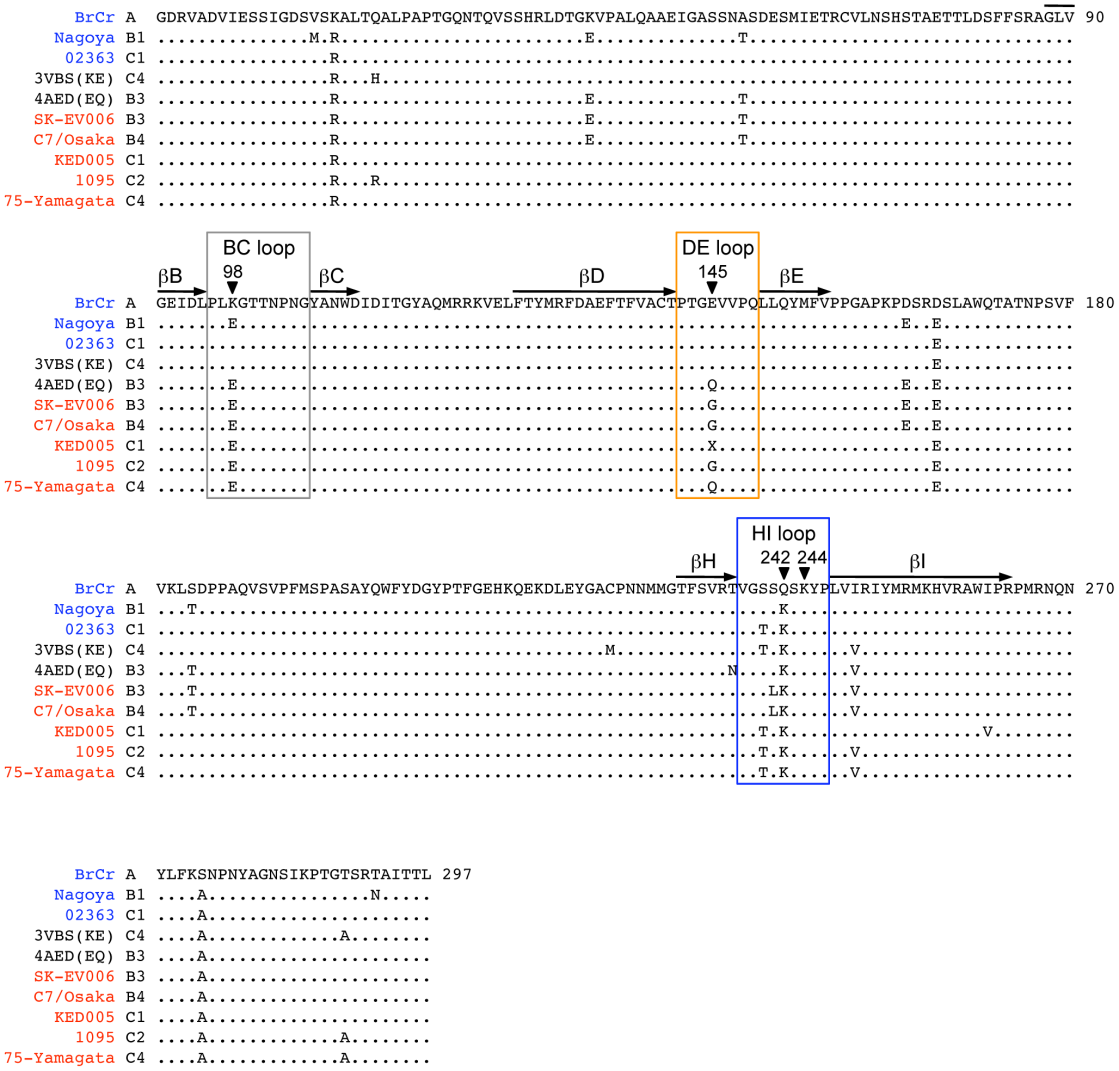
Alignment of EV71 VP1 sequences. The names of the EV71-PB and EV71-non-PB strains are in red and blue, respectively. 4AED (EV71-EQ) and 3VBS (EV71-KE) in black are presumably PB and non-PB viruses, respectively. The names are followed by their subgenogroups. The BC, DE, and HI loops (boxed) and the β barrels (shown with arrows) are defined as in reference 4. The amino acids where mutations were introduced are indicated by arrowheads. The conserved amino acids are shown with dots. The amino acid at VP1-145 (X) of EV71-KED005 could not be determined because there was a mixture of sequences at his position.

Recent crystal structures of the mature EV71 virion [2], [3], [4] reveal that VP1-145 is located on the virus surface within the VP1-DE loop, which connects the βD and βE barrels, on a mesa that surrounds the viral five-fold axis of symmetry (Figures 3 and 4). Based on the observation that virus binding requires negatively-charged sulfated tyrosines within the N-terminal region of PSGL-1 [26]— the site of virus interaction [12]— we suspected that the binding might depend on positively-charged amino acid side chains exposed on the virus surface. Analysis of the electrostatic surface properties of the EV71 crystal structures with UCSF Chimera software showed the region of positive electrostatic potential (colored blue) around the five-fold axis contributed by VP1-242K and VP1-244K (Figure 3, A and B) [4]. Interestingly, we found that these lysine residues, VP1-242K and VP1-244K, are in close proximity to VP1-145 (Figure 3, B and C). In particular, VP1-145 in the DE loop makes close contact with VP1-244K in the HI loop from an adjacent protomer (Figure 3D).

Two crystal structures of the EV71 capsid are available. Plevka *et al.* [2], [3] reported the structure of an EV71 isolate with Q at VP1-145, presumably a PB virus (Protein Data Bank Accession No. 4AED) (Figure 3, A–D, top; Figure 4). In contrast, Wang *et al.* [4] reported the structure of an isolate with E at VP1-145, presumably a non-PB isolate (Protein Data Bank Accession No. 3VBS) (Figure 3, A–D, bottom; Figure 4). Interestingly, the orientation of the basic side chain of VP1-244 is markedly different in the two structures (Figure 3, C and D); in the likely PB virus, the VP1-244K side chain projects outward, with the positively charged ε-amino group highly exposed on the virus surface; in the presumed non-PB virus, the lysine side chain is oriented toward VP1-145E which provides negatively charged patches (Figure 3B, arrowheads), and the positively charged group is less exposed. A homology model generated with SWISS-MODEL (http://swissmodel.expasy.org/) [31], [32], [33] revealed that VP1-145E to G or Q substitution of 3VBS (presumed non-PB) abolished negatively charged patches, whereas VP1-145Q to E substitution of 4AED (presumed PB) generated negatively charged patches (not shown). These results suggested that VP1-145 might regulate the PB phenotype by modulating the orientation of the conserved lysine residues, particularly the orientation of VP1-244.

### VP1-244K is essential for virus binding to PSGL-1

**Figure 5:**
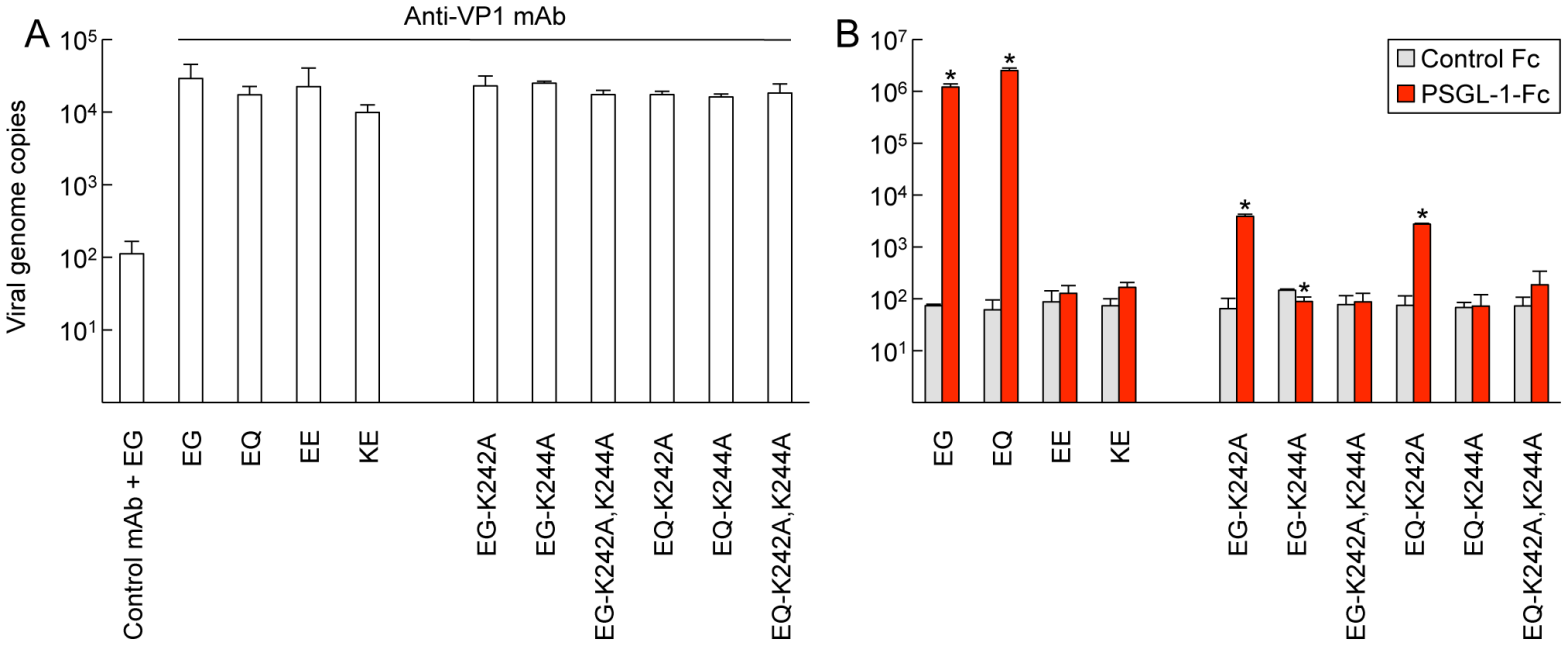
EV71–PSGL-1 binding assay using real-time RT-PCR. Viruses (5×10^7^ viral genomes) were incubated with anti-VP1 mAb or PSGL-1-Fc and collected with protein G beads. Precipitated viruses were analyzed by real-time RT-PCR as described in Materials and Methods. Viral genome copies are expressed as the mean, and error bars indicate s. d. of three independent experiments. (A) Viruses were precipitated with anti-VP1 mAb to show the presence of virion in the sample. The amount of virus precipitated with nonspecific isotype control was considered as background binding (left). (B) Viruses were precipitated with PSGL-1-Fc. A control Fc chimeric protein (CTLA-4-Fc) was used as a negative control. Asterisks indicate a significant difference in specific binding to PSGL-1-Fc (*P*<0.01). Although there was a significant difference in EG-K244A, we consider it biologically meaningless.

To test the role of these lysines, we introduced VP1-K242A and/or VP1-K244A substitutions into two PB viruses, EV71-1095-EG and EV71-1095-EQ. We found that when these mutant viruses were generated in RD cells, the recovered viruses had a number of undesired mutations at VP1-145. We reasoned that, because picornavirus replication is highly error prone, fewer mutations might arise in RNA-transfected CHO-K1 cells, which do not support multiple rounds of viral replication [14]. Because only limited amounts of virus could be obtained in this way, we established a highly sensitive EV71–PSGL-1-binding assay based on real-time quantitative RT-PCR. To detect viral RNA within particles bound to soluble PSGL-1, it is important to note that attachment to PSGL-1 does not cause to release of RNA from virion [34]. Virus-containing cell culture supernatants of RNA-transfected CHO-K1 cells were used directly for co-precipitation assay with PSGL-1-Fc at 4°C, RNA genomes were released from precipitated virus by heating at 95°C [35], and genome numbers were measured by real-time RT-PCR [36]. Genome numbers in the input viral supernatants were measured by immunoprecipitation of supernatants with anti-VP1 mAb (Figure 5A).

When the assay was performed with previously characterized PB viruses (EV71-1095-EG or EV71-1095-EQ) and non-PB viruses (EV71-1095-EE and EV71-1095-KE), the results were consistent with those obtained in Figure 1: for the PB viruses, numerous viral genomes were precipitated with PSGL-1-Fc but not with control Fc protein (Figure 5B, left); in contrast, for the non-PB viruses, few genomes were precipitated with either protein. Having thus determined that the assay could measure virus binding to PSGL-1, we tested binding of the VP1-K242A and VP1-K244A mutants. The VP1-K242A mutation permitted a low level of residual binding, but the total binding was reduced more than 100 fold. Both the VP1-K244A mutation and the double mutation of VP1-K242A and VP1-K244A abolished PSGL-1 binding. Thus, although lysine residues at VP1-242 and VP1-244 both contribute to binding, only the lysine at VP1-244 — which makes closer contact with VP1-145— is absolutely essential.

## Discussion

The results we report here demonstrate that a single amino acid, VP1-145, is the critical determinant of EV71 tropism for PSGL-1. We found that the presence of a G or Q residue at this position permitted viruses belonging to a variety of genogroups to bind PSGL-1, whereas viruses with E at this position did not bind PSGL-1. Similarly, viruses with G or Q, but not E at VP1-145, were found to replicate in Jurkat T lymphocytes, supporting the idea that VP1-145, by controlling interaction with PSGL-1, plays an important role in virus tropism for human leukocytes.

Sulfated tyrosines in the PSGL-1 N-terminus are critical for interaction with P-selectin, and crystal structure analysis demonstrates that two sulfated tyrosines in the PSGL-1 N-terminus interact directly with positively charged residues in the P-selectin lectin domain [37]. Interaction of the HIV envelope glycoprotein gp120 with the coreceptor CCR5 depends a sulfation of a tyrosine residue within CCR5, and structural analysis suggests that this tyrosine interacts with a positively charged residue of gp120 [38]. Based on the observation that EV71 binding depends on tyrosine sulfation of PSGL-1 we had expected that binding must involve positively charged residues on the virus surface, and were somewhat surprised to identify VP1-145 G and Q as critical. However, VP1-145, which is located on a mesa surrounding the five-fold axis, is in close proximity to two highly conserved lysine residues; we find that these lysine residues— in particular, VP1-244, which is in close contact with VP1-145— are also required for binding to PSGL-1. Based on the available crystal structures of viruses with Q versus E at VP1-145, we propose that VP1-145 modulates the orientation of lysine VP1-244, and thus regulates exposure of the positively charged lysine side chain.

Why does EV71 use VP1-145, rather than the lysines at VP1-242 or VP1-244, to control the PSGL-1-binding phenotype? One possible explanation is that these lysines, which are conserved in 1618 (VP1-242) and 1619 (VP1-244) out of 1623 of the EV71 isolates sequenced in GenBank, serve another function critical for virus interaction with host cells. Recently, Tan *et al.* [13] showed that EV71 binds to heparan sulfate on the cell surface, and suggested that heparan sulfate may bind to positively charged amino acids (including the lysines at VP1-242 and 244, as well as an arginine at VP1-161), that form a cluster around the five-fold symmetry axis. Interaction of coxsackievirus A9 with heparan-sulfate proteoglycan has been shown to depend on a similar cluster of charged residues (in this case, 5 copies of a single arginine) at the five-fold axis [39]. If interaction with heparan sulfate is critical for EV71 infection, or for persistence of EV71 in the human population, the virus may have evolved an indirect mechanism, using VP1-145, to control PSGL-1 interaction.

**Figure 6:**
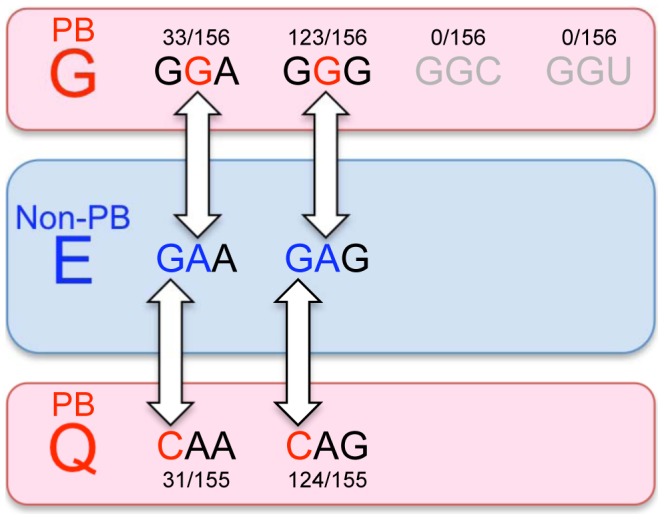
The evolution of EV71-PB and EV71-non-PB . The amino acids at VP1-145 and their codons are shown. The numbers above or below the codons indicate their frequency in GenBank. EV71 with VP1-145G or VP1-145Q are the PSGL-1-binding (PB) phenotype and the minor populations in GenBank (red round rectangles). EV71 with VP1-145E is the PSGL-1-nonbinding (non-PB) phenotype and the major population in GenBank (blue round rectangles). The phenotypic change between PSGL-1-binding and PSGL-1-nonbinding can occur easily, because only one nucleotide substitution changes amino acid (white arrows). The amino acid change between G and Q would not occur easily, as their codons have two nucleotide differences. Neither GGC nor GGU was found to encode VP1-145G in GenBank.

We found that more than 80% of EV71 sequences in GenBank had an E residue at VP-145 (non-PB), and approximately 20% had G or Q (PB). When we examined the specific sequences encoding VP1-145 we found that single nucleotide changes in the codons for E (GAA, GAG) lead to codons for G (GGA, GGG) and Q (CAA, CAG) (Figure 6). Thus, single nucleotide changes are sufficient for replacement of E with Q or G (or *vice versa*), and for viruses to gain or lose the capacity to bind PSGL-1. As there are two nucleotide differences between the codons for G and Q, direct conversion between G and Q is less likely to occur; this suggests that PB viruses in the database are more likely to have derived from non-PB viruses with E at VP1-145 than to have evolved from other PB viruses. It is interesting to note that although four codons are available to specify G, two G codons that cannot be directly obtained from E codons are not seen at VP1-145. Based on an analysis of the ratio of non-synonymous to synonymous substitutions in the capsid proteins of EV71, VP1-145 has been identified as a major site of positive selection [29], [40], [41]. Thus, the rapid amino acid change and polymorphism at VP1-145 may contribute to viral fitness *in vivo* by changing the cell tropism of EV71 variants.

In two recent studies, EV71 sequences from patients were examined in an effort to identify genetic markers of virulence; in both studies VP1-145 was associated with disease severity, and VP1-145E was associated with milder disease [42], [43]. Although these studies are not definitive, it is tempting to speculate that isolates with G or Q at this position may be virulent because of their capacity to bind PSGL-1. However, mutations at VP1-145 from G or Q to E have been shown to confer mouse adaptation and virulent phenotypes in different mouse models [44], [45], [46]; because mouse PSGL-1 does not bind EV71 [12], VP1-145 may also influence virulence by mechanisms that do not involve interactions with PSGL-1.

A major event in the entry process is uncoating, release of viral RNA from the capsid into the cytoplasm. EV71 interaction with SCARB2, particularly under acidic conditions, has been shown to convert the native virion to a 135S A particle [30], [34], which is considered to be an intermediate in the uncoating process. In contrast, EV71 interaction with PSGL-1 does not result in formation of A particles [30], [34]. Capsid residues important for interaction with SCARB2 map to the cleft or canyon that surrounds the five-fold mesa [30], suggesting that SCARB2— like the poliovirus receptor, which also induces formation of A particles [47]— binds within the canyon. The capsid residues we find to be important for binding to PSGL-1 are remote from the canyon, and cluster at the five-fold axis, surrounding a small cavity remote from the canyon. It is possible that the thin N-terminal region of PSGL-1 inserts into this cavity, and that the interaction is stabilized by interactions between the sulfated tyrosines and lysine residues at VP1-242 and -244. The acidic side chain of VP1-145E may repel the sulfated tyrosines of PSGL-1. Analysis using cyro-electron microscopy will be important to clarify the contact sites between EV71 and PSGL-1, and crystal structures of EV71-EG and -EE (in addition to EV71-EQ and -KE) will provide a more detailed picture of the role of the lysine residues.

Because PSGL-1 is involved in leukocyte migration, cytokine production, and regulation of immune responses [48], [49], [50], [51], [52], virus interaction with PSGL-1 on leukocytes may be important for dissemination within the host, for modulation of antiviral host responses, or for the excessive inflammatory cytokine production seen in patients with severe disease. The work we describe here indicates that EV71 can rapidly change its avidity for PSGL-1, and its tropism for PSGL-1-expressing leukocytes, as the consequence of a single amino acid change at VP1-145. Our results are significant for understanding of virus-host interactions, viral evolution, and pathogenesis.

## Materials and Methods

### Cells

Jurkat cells were obtained from Riken Cell Bank and cultured in RPMI-1640 medium (Sigma) supplemented with 10% fetal bovine serum (FBS). RD cells obtained from the US Centers for Disease Control were maintained in DMEM (Sigma) supplemented with 10% FBS. CHO-K1 cells were maintained in F-12 nutrient mixture (Ham) (Life technologies) supplemented with 10% FBS.

### Viruses

We used eight representative EV71 isolates characterized in our previous study [12] (Table 1). BrCr was originally isolated from a meningitis patient [53]. SK-EV006 and C7/Osaka were isolated from a patient with fatal encephalitis [54]. Other strains were isolated from HFMD patients without severe symptom [55], [56], [57], [58]. Stocks of clinical isolates were prepared in RD or Vero cells. For most experiments, virus produced by RNA-transfected cells was amplified by growth in RD cells; supernatants were titered, and clarified by centrifugation in a microfuge at 15,000 rpm for 3 min just before use. For the experiments shown in Figure 2, viruses were concentrated by ultracentrifugation, as follows: culture supernatant was centrifuged at 10,000 rpm in a Beckman SW32Ti rotor for 1 h to precipitate cell debris; the supernatant was then spun in the same rotor at 32,000 rpm for 2 h, and the resulting pellet was resuspended in phosphate-buffered saline (+) at 4°C overnight. For experiments shown in Figure 5, virus was produced in RNA-transfected CHO cells; debris was pelleted in a microfuge at 15,000 rpm for 3 min, and the supernatant was used for binding experiments. In all cases, viral titers were determined by a microtitration assay using 96-well plates and RD cells as previously described [56]. Briefly, 10 wells were used for each viral dilution and the viral titers were expressed as 50% cell culture infectious dose (CCID_50_).

### Monoclonal antibodies and recombinant proteins

We used the EV71-specific mAb MA105 (mouse IgG_2b_) (Y. Tano *et al.*, unpublished). The mAb to human PSGL-1 (KPL1; mouse IgG_1_) was purchased from BD Biosciences. For negative controls, mouse IgG_1_ (MOPC-21) and IgG_2b_ (MOPC-141) were purchased from BioLegend and Sigma, respectively. Soluble recombinant forms of human proteins fused to the Fc region of human IgG_1_ (PSGL-1-Fc and CTLA-4-Fc) were purchased from R&D Systems.

### Sequence analysis of the genomes of the EV71 strains

The genomic sequence of EV71 was determined as described previously [27]. Briefly, we extracted viral genomic RNA from the culture supernatant of infected RD cells. We performed RT-PCR preparation of DNA fragments for direct DNA sequencing. The 5′ and 3′ ends of the viral genome were sequenced using the conventional RACE methods.

### Construction of the infectious viral cDNA clones

Viral cDNA was reverse transcribed using CDS III/3′ PCR Primer (5′-ATTCTAGAGGCCGAGGCGGCCGACATG-dT(30)-VN-3′) (Takara) and Super Script II polymerase (Life technologies). Full genomic cDNA was amplified by PCR using the primers in Table S2. For cloning of the full genomic cDNA, we introduced the multi cloning sites into the pBR322 plasmid (Takara). The *Eco*RI-*Bsm*I fragment of pBR322 was replaced with 5′- GAATTCCTTAAGCTCGAGTCTAGACCCGGGGGATCCGTGCACAGGCCTCG -3′ (*Eco*RI+*Afl*II+*Xho*I+*Xba*I+*Sma*I+*Bam*HI+*Apa*LI+*Stu*I+cg) to produce pBR322Y. The full genomic cDNA was cloned into pBR322Y and *Escherichia coli* strain XL10-Gold (Agilent technologies) was used for the preparation of the plasmids. The nucleotides different from those obtained from direct sequencing of RT-PCR products were corrected by site directed mutagenesis using PCR or by replacing them with the restriction enzyme-digested DNA fragment from a plasmid with the correct nucleotide sequence. We used the genomic RNA of C7/Osaka, Nagoya, 1095, 02363, and 75-Yamagata strains of EV71 as the template for RT-PCR and named the resultant plasmids as pBREV71-C7/Osaka-EG, pBREV71-Nagoya-EE, pBREV71-1095-EG, pBREV71-02363-KE, pBREV71-75-Yamagata-EQ, respectively. The mutations were introduced into the plasmids by site directed mutagenesis using PCR. The primers used for mutagenesis are provided in Table S3. The plasmids and viruses with mutations at VP1-98 and/or VP1-145 were named as shown in Figure 1A.

### Generation of viruses from the infectious viral cDNA clones

We generated viruses from infectious viral cDNA clones as described previously [44]. Briefly, RNA transcripts of EV71 mutants were obtained using a MEGAscript T7 kit (Life technologies) or RiboMAX large scale RNA production system-T7 (Promega) with linearized DNA of the infectious EV71 clones as the template. RNA transcripts were transfected into a monolayer of RD cells in six-well plates using a Lipofectamine 2000 reagent (Life technologies) or 2 mg ml^−1^ of polyethyleneimine “MAX” (MW 40,000) (Polysciences) [59], followed by incubation at 37°C. The medium was replaced with fresh medium 4 h after transfection. The transfected cells and supernatants were freeze-thawed three times at 24 h post-transfection. Before use in experiments, the recovered viruses were amplified once in fresh RD cells, and the sequence of the whole capsid region was confirmed by direct sequencing of RT-PCR products.

CHO-K1 cells were used to prepare viruses for the highly sensitive EV71–PSGL-1-binding assay using real-time RT-PCR. CHO-K1 cells were seeded at 2.5×10^5^ cells per 2.5 ml in a 6-well plate 18 h before transfection. Just before transfection, the medium was replaced with 2.5 ml of F-12 nutrient mixture without FBS after washing the cells. Five µg of RNA transcripts was transfected according to the manufacturer's for Lipofectamine 2000 (Life technologies), expect for using 10 µl of 2 mg ml^−1^ polyethyleneimine “MAX” (MW 40,000) (Polysciences) instead of Lipofectamine 2000. The medium was replaced with 1.2 ml of fresh medium with 10% FBS 4 h after transfection. The transfected cells and supernatants were freeze-thawed three times at 24 h post-transfection. The supernatant was used for the binding assay.

### EV71–PSGL-1-Fc binding assay by immunoprecipitation and western blotting

The EV71–PSGL-1-Fc binding assay [12] was performed with minor modification. Briefly, Dynabeads protein G (Life technologies) and 1 µg of chimeric Fc proteins were diluted in 300 µl of immunoprecipitation buffer (20 mM Tris-Cl, 135 mM NaCl, 1% Triton X-100, 10% glycerol; pH 7.4) and incubated for 1 h at 4°C. The beads were washed once. Viruses concentrated by ultracentrifugation (0.5 µg VP1 protein in SDS-PAGE analysis) were added and incubated in immunoprecipitation buffer for an additional 1 h. We washed the beads and subjected the immunoprecipitates to 12.5% SDS-PAGE. For Western blotting, proteins were transferred onto nitrocellulose membranes and blotted with anti-EV71 VP1 mAb MA105.

### EV71–PSGL-1-Fc binding assay by immunoprecipitation and real-time RT-PCR

Ten µl of Dynabeads protein G (Life Technologies) and 0.5 µg of chimeric Fc proteins were diluted in 10 µl of immunoprecipitation buffer and 80 µl of F-12 nutrient mixture (Life technologies) with 10% FBS and incubated for 1 h at 4°C. The beads were washed once and incubated with 50 µl of the diluted supernatant of CHO-K1 cell culture with 5×10^7^ copies of the EV71 RNA genome, 10 µl of immunoprecipitation buffer, and 40 µl of F-12 nutrient mixture (Life technologies) with 10% FBS for 1 h. The beads were washed five times with immunoprecipitation buffer and suspended in 50 µl of DEPC-treated water. The immunoprecipitates were incubated at 95°C for 5 min to release the virion RNA [35]. Real-time RT-PCR was performed as described previously by Johnsson *et al.* [36] with modifications. Five µl of viral RNA was assayed in a 20 µl reaction mixture using a Power SYBER Green RNA-to-Ct 1-step Kit (Life technologies) with primers EnteroFw and EnteroRev (final 100 nM each) [36]. The mixtures were subjected to real-time RT-PCR, consisting of a reverse transcription step at 42°C for 30 min followed by 40 cycles of 95°C for 3 s and 60°C for 30 s. The results were analyzed with 7500 Fast Real-Time PCR System (Life technologies). Viral RNA of EV71-1095-EG was used for quantification of copy number.

### Virus infection assays

RD cells (2×10^5^ cells per 200 µl in a 48-well plate) were inoculated with viruses at 1 CCID_50_ per cell for 1 h, washed, and incubated in 400 µl of the medium at 37°C. Jurkat cells (4×10^4^ cells) were inoculated with viruses at 1 CCID_50_ per cell for 1 h, washed, and incubated in 400 µl of the medium in a 48-well plate at 34°C. For mAb inhibition, the cells were pretreated with 10 µg ml^−1^ mAb in 100 µl of the medium for 1 h, washed, and maintained with 10 µg ml^−1^ mAb in 400 µl of the medium. The culture supernatants and infected cells were subjected to three cycles of freeze-thawing before titration.

### Structural analysis

Crystal structures of Protein Data Bank Accession No. 4AED and 3VBS were used for presumed EV71-PB and non-PB, respectively. Molecular graphics and analyses were performed with the UCSF Chimera package [60].

### Statistical analysis

We carried out all infection assays in triplicate and compared the mean viral titers using Student's *t*-test (two-tailed). *P* values of <0.01 were considered statistically significant.

## Supporting Information

Figure S1 EV71–PSGL-1 binding assay using real-time RT-PCR. Viruses (5×10^7^ viral genomes) were incubated with anti-VP1 mAb or PSGL-1-Fc and collected with protein G beads. Precipitated viruses were analyzed by real-time RT-PCR as described in Materials and Methods. Viral genome copies are expressed as the mean, and error bars indicate s. d. of three independent experiments. (A) Viruses were precipitated with anti-VP1 mAb to show the presence of virion in the sample. The amount of virus precipitated with nonspecific isotype control was considered as background binding (left). (B) Viruses were precipitated with PSGL-1-Fc. A control Fc chimeric protein (CTLA-4-Fc) was used as a negative control. Asterisks indicate a significant difference in specific binding to PSGL-1-Fc (*P*<0.01).
(TIF)

Table S1 Codons and amino acids at VP1-98 and VP1-145 in 1702 EV71 sequences found in GenBank.
(XLSX)

Table S2 Primers for PCR amplification of EV71 genomes.
(DOCX)

Table S3 Primers for substitution.
(DOCX)
