# Supplementary figures and images for: Enterovirus 71 Binding to PSGL-1 on Leukocytes: VP1-145 Acts as a Molecular Switch to Control Receptor Interaction
Source: PLoS Pathog. 2013 Jul 25;9(7):e1003511. doi: 10.1371/journal.ppat.1003511 (PMC3723564; doi:10.1371/journal.ppat.1003511)

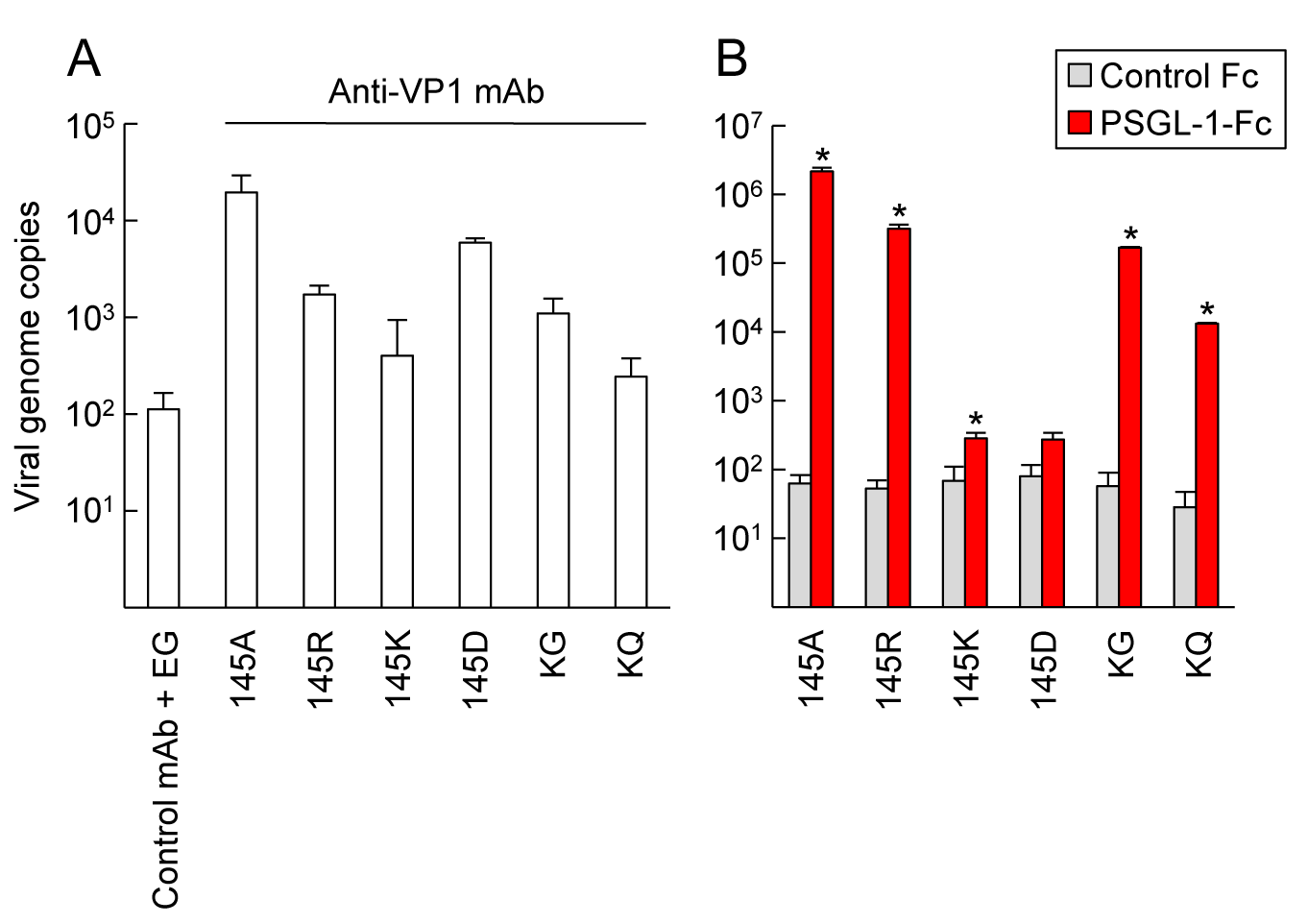

Supplement: Figure S1 — EV71–PSGL-1 binding assay using real-time RT-PCR. Viruses (5×10^7^ viral genomes) were incubated with anti-VP1 mAb or PSGL-1-Fc and collected with protein G beads. Precipitated viruses were analyzed by real-time RT-PCR as described in Materials and Methods. Viral genome copies are expressed as the mean, and error bars indicate s. d. of three independent experiments. (A) Viruses were precipitated with anti-VP1 mAb to show the presence of virion in the sample. The amount of virus precipitated with nonspecific isotype control was considered as background binding (left). (B) Viruses were precipitated with PSGL-1-Fc. A control Fc chimeric protein (CTLA-4-Fc) was used as a negative control. Asterisks indicate a significant difference in specific binding to PSGL-1-Fc (P<0.01). (TIF) [file ppat.1003511.s001.tif]
