# Supplementary material for: Enterovirus 71 Binding to PSGL-1 on Leukocytes: VP1-145 Acts as a Molecular Switch to Control Receptor Interaction
Source: PLoS Pathog. 2013 Jul 25;9(7):e1003511. doi: 10.1371/journal.ppat.1003511 (PMC3723564; doi:10.1371/journal.ppat.1003511)
Supplement: Table S2 — Primers for PCR amplification of EV71 genomes. (DOCX) [file ppat.1003511.s003.docx]

**Table S2. Primers for PCR amplification of EV71 genomes.**

| Construct | S/A^1)^ | Site | Sequence (5'-3')^2)^ |
| --- | --- | --- | --- |
| EV71-C7/Osaka | S | *Bam*HI | ttaggatcc*taatacgactcactata*ggTTAAAACAGCCTGTGGGTTGCACCCAC |
| EV71-Nagoya | S | *Afl*II | ttacttaag*taatacgactcactata*ggTTAAAACAGCCTGTGGGTTGTTCCCAC |
| EV71-1095 | S | *Eco*RI | ttagaattc*taatacgactcactata*ggTTAAAACAGCCTGTGGGTTGCACCCAC |
| EV71-02363 | S | *Eco*RI | ttagaattc*taatacgactcactata*ggTTAAAACAGCCTGTGGGTTGTACCCAC |
| EV71-75-Yamagata | S | *Xba*I | ttatctaga*taatacgactcactata*ggTTAAAACAGCCTGTGGGTTGCACCCAC |
| EV71-C7/Osaka  EV71-Nagoya | A | *Stu*I | aaaaggccttttttttttttttttttttttttttGCTATTCTGGTTATAACAAATTTACCCCCAC |
| EV71-1095 | A | *Bam*HI | aaaggatcctttttttttttttttttttttttttGCTATTCTGGTTATAACAAATTTACCCCCAC |
| EV71-02363  EV71-75-Yamagata | A | *Sma*I | aaacccgggtttttttttttttttttttttttttGCTATTCTGGTTATAACAAATTTACCCCCAC |

^1)^ S, sense; A, antisense

^2)^ Restriction endonuclease recognition sites are underlined. T7 promoter sequences are indicated by italics. Nucleotides corresponding to the virus genome are indicated by uppercase letters.
