# Supplementary material for: Enterovirus 71 Binding to PSGL-1 on Leukocytes: VP1-145 Acts as a Molecular Switch to Control Receptor Interaction
Source: PLoS Pathog. 2013 Jul 25;9(7):e1003511. doi: 10.1371/journal.ppat.1003511 (PMC3723564; doi:10.1371/journal.ppat.1003511)
Supplement: Table S3 — Primers for substitution. (DOCX) [file ppat.1003511.s004.docx]

**Table S3. Primers for substitution.**

| Construct^1)^ | S/A^2)^ | Sequence (5'-3')^3)^ |
| --- | --- | --- |
| C7/Osaka VP1-98K (g2734a) | S | ccctcttAagggtaccactaatccaaatgg |
|  | A | gtaccctTaagagggagatctatctctcct |
| C7/Osaka VP1-145Q (gg2875-6ca) | S | tactggtCAggttgttccacaattactcca |
|  | A | aacaaccTGaccagtaggagtgcacgcaac |
| C7/Osaka VP1-145E (g2876a) | S | tactggtgAggttgttccacaattactcca |
|  | A | aacaaccTcaccagtaggagtgcacgcaac |
| Nagoya VP1-98K (g2734a) | S | ccctcttAagggtactactaacccaaatgg |
|  | A | gtaccctTaagagggaggtctatctcccct |
| Nagoya VP1-145G (a2876g) | S | tactggcgGggttgttccacaattactcca |
|  | A | aacaaccCcgccagtaggggtgcacgcaac |
| Nagoya VP1-145Q (g2875c) | S | tactggcCaggttgttccacaattactcca |
|  | A | aacaacctGgccagtaggggtgcacgcaac |
| 1095 VP1-98K (g2731a) | S | ccctcttAaaggcacaaccaacccgaatgg |
|  | A | gtgccttTaagagggaggtctatctctcca |
| 1095 VP1-145Q (gg2872-3ca) | S | taccgggCAggttgttccgcaattgctcca |
|  | A | aacaaccTGcccggtaggcgtgcacgcaac |
| 1095 VP1-145E (g2873a) | S | taccggggAggttgttccgcaattgctcca |
|  | A | aacaaccTccccggtaggcgtgcacgcaac |
| 1095 VP1-242A (aa3163-4gc) | S | tcgGCatccaagtacccattagtggtcaggattt |
|  | A | tgggtacttggatGCcgaggtccctacagtccgca |
| 1095 VP1-244A (aa3169-70gc) | S | tcgaaatccGCgtacccattagtggtcaggattt |
|  | A | tgggtacGCggatttcgaggtccctacagtccgca |
| 1095 VP1-242A, 244A (aa3163-4gc, aa3169-70gc) | S | tcgGCatccGCgtacccattagtggtcaggattt |
|  | A | tgggtacGCggatGCcgaggtccctacagtccgca |
| 1095 VP1-145A (g2873c) | S | taccggggCggttgttccgcaattgctcca |
|  | A | aacaaccGccccggtaggcgtgcacgcaac |
| 1095 VP1-145D (gg2873-4ac) | S | taccggggACgttgttccgcaattgctcca |
|  | A | aacaacGTccccggtaggcgtgcacgcaac |
| 1095 VP1-145R (g2872c) | S | taccgggCgggttgttccgcaattgctcca |
|  | A | aacaacccGcccggtaggcgtgcacgcaac |
| 1095 VP1-145K (gg2872-3aa) | S | taccgggAAggttgttccgcaattgctcca |
|  | A | aacaaccTTcccggtaggcgtgcacgcaac |
| 02363 VP1-98E (a2731g) | S | ccctctcGaaggcacaaccaacccgaatgg |
|  | A | gtgccttCgagagggaggtctatctctcca |
| 02363 VP1-145G (a2873g) | S | caccggggGggttgtcccacaattgctcca |
|  | A | gacaaccCccccggtgggtgtgcatgcaac |
| 02363 VP1-145Q (g2872c) | S | caccgggCaggttgtcccacaattgctcca |
|  | A | gacaacctGcccggtgggtgtgcatgcaac |
| 75-Yamagata VP1-98K (g2728a) | S | ccctcttAaaggcacaactaacccaaatgg |
|  | A | gtgccttTaagagggagatctatctctcca |
| 75-Yamagata VP1-145Q (ca2869-70gg) | S | caccgggGGagttgtcccacaattgctcca |
|  | A | gacaactCCcccggtgggtgtgcacgcaac |
| 75-Yamagata VP1-145E (c2869g) | S | caccgggGaagttgtcccacaattgctcca |
|  | A | gacaacttCcccggtgggtgtgcacgcaac |

^1)^The nucleotide mutations and their positions were indicated in parentheses.

^2)^S, sense; A, antisense.

^3)^Substituted nucleotides are indicated by uppercase letters.
